# Tubulo-villous adenoma of the appendix: A case report and review of the literature

**DOI:** 10.1016/j.ijscr.2019.06.061

**Published:** 2019-07-16

**Authors:** Giuseppe Evola, Giovambattista Caruso, Sebastiano Caramma, Giovanni Dapri, Carmela Spampinato, Carlo Reina, Giuseppe Angelo Reina

**Affiliations:** a General and Emergency Surgery Department, Garibaldi Hospital, Catania, Italy; b General Surgery Department, San Salvatore Hospital, Paternò, Catania, Italy; c Department of Gastrointestinal Surgery, Saint-Pierre University Hospital, Brussels, Belgium

**Keywords:** Tubulo-villous adenoma, Appendiceal neoplasms, Acute appendicitis, Risk factors, Appendectomy, Case report

## Abstract

**Introduction:**

Tubulo-villous adenoma is a rare benign appendiceal neoplasm often asymptomatic with the most clinical manifestation that resembles acute appendicitis. Pre-operative diagnosis is difficult by its rarity and the absence of typical symptoms. Adequate treatment is surgical resection.

**Presentation of case:**

A 69-year-old male was admitted to the Emergency Department with a two-day history of abdominal pain associated with constipation. Abdominal examination revealed abdominal pain localized, at deep palpation, in the right iliac fossa and in hypogastrium without obvious muscle guarding or rebound tenderness. Laboratory tests showed a normal white blood cell count with 82.3% neutrophils and high C-reactive protein level. After a negative abdominal ecography, the patient was evaluated by abdominal computed tomography, which revealed acute appendicitis. The patient was submitted to surgery and open appendectomy was performed. The post-operative course was uneventful and the patient was discharged on the 5^th^ post-operative day.

**Discussion:**

Acute appendicitis may be a clinical manifestation of a benign appendiceal neoplasm. Pre-operative radiological investigations not always are useful for an early diagnosis that is mandatory because of the potential risk of malignant degeneration. Appropriate treatment of acute appendicitis is debated: some surgeons suggest operative treatment, but others advocate for non-operative management. In our case the patient was submitted to surgery avoiding the risk of diagnostic delay of neoplasm.

**Conclusion:**

Appendiceal tubulo-villous adenoma is a rare neoplasm difficult to diagnose and suspect because of lack of pathognomonic symptoms and specific diagnostic signs. Acute appendicitis is the most common clinical presentation. Appendectomy is the appropriate treatment.

## Highlights

• Tubulo-villous adenoma is a rare benign appendiceal neoplasm.
• This neoplasm is often asymptomatic and occasionally discovered at surgery.
• Acute appendicitis is the most common clinical presentation.
• Pre-operative diagnosis, even with the help of radiological imaging, is difficult.
• Appendectomy is considered the correct treatment.

## 1 Introduction

Appendiceal neoplasms are uncommon tumors, represent less than 0.5% of all gastrointestinal malignancies [1] and are found in approximately 1% of appendectomy specimens [2]. The incidence of these neoplasms may have been underreported in the past. The most common appendiceal tumors are epithelial neoplasms and neuroendocrine tumors (NETs), others rarety are lymphoma, metastases, neuroectodermal and nerve sheath tumors, mesenchymal tumors and Kaposi sarcoma [3]. Benign neoplasms are often asymptomatic and incidentally discovered at clinical examination, at time of surgery or pathologic evaluation after appendectomy; malignant neoplasms may cause symptoms related to regional involvement, peritoneal spread or metastatic disease. The most frequent initial manifestation of appendiceal neoplastic lesions is acute appendicitis, reported in 30–50% of patients and more commonly in NETs than in epithelial neoplasms [2]. We report a case of acute appendicitis caused by non-mucinous tubulo-villous adenoma. The present work has been reported in accordance with the Surgical Case Reports (SCARE) criteria [4].

## 2 Presentation of case

**Fig. 1:**
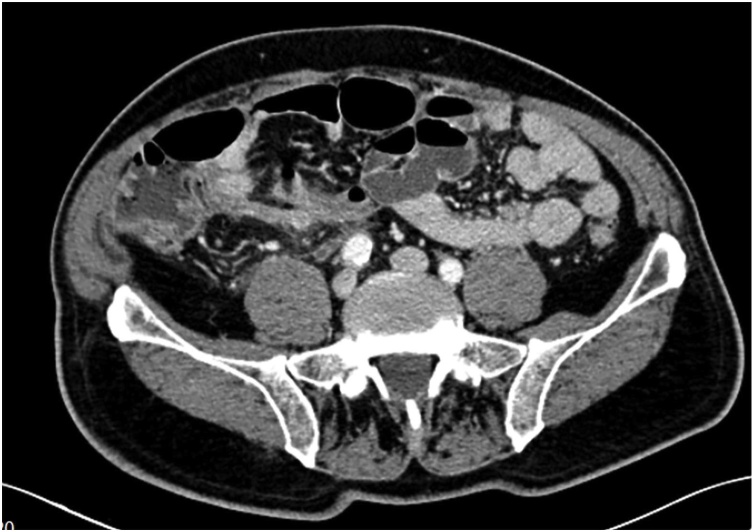
Abdominal CT scan showing acute appendicitis.

**Fig. 2:**
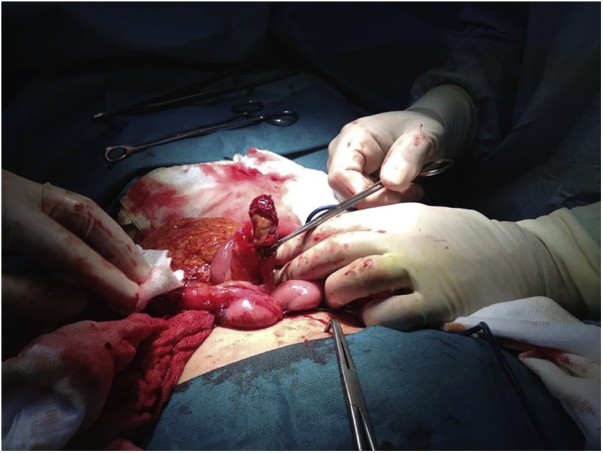
Perforated acute appendicitis: perioperative findings.

**Fig. 3:**
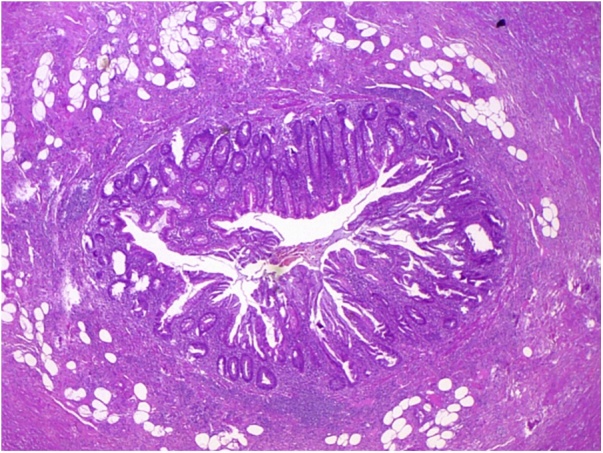
Photomicrograph section of thr appendicular lumen (haematoxylin ans eosin, original magnification ×10).

**Fig. 4:**
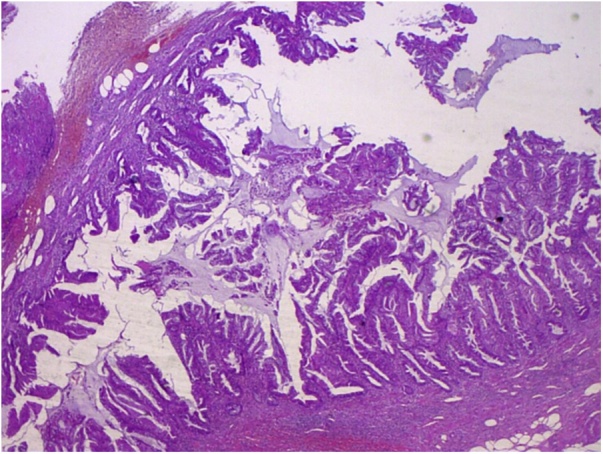
Photomicrograph tubulo-villous adenoma of the appendix (haematoxylin and eosin, original magnification ×10).

A 69-year-old Caucasian male with a medical history of hypertension, diabetes mellitus, chronic ischemic heart disease and chronic obstructive bronchopathy, was admitted to the Emergency Department with a two-day history of abdominal pain associated with costipation. Abdominal examination revealed distension with tympanic percussion and the presence of abdominal pain localized, at deep palpation, in the right iliac fossa and in hypogastrium without obvious muscle guarding or rebound tenderness. The patient denied any fever, nausea, vomiting or loss of appetite. Laboratory tests showed a normal white blood cell count of 9.9 × 10^3^/μL (with 82.3% neutrophils), C-reactive protein (CRP) level of 326.80 mg/L (reference range <7.5 mg/L), hyperglycemia (324 mg/dL), normal levels of hemoglobin (14.30 g/dL) and hematocrit (42.20%), high level of fibrinogen (1020 mg/dL). Abdomen Rx showed dilated bowel loops in the mid-lower abdominal quadrants. After a negative abdominal ecography, the patient was evaluated by abdominal computed tomography (CT), which revealed the presence of a post-ileal appendix with mild wall thickening and periappendiceal inflammatory changes consistent in acute appendicitis (Fig. 1), without evidence of endoluminal neoplasia. The patient during pre-operative work-up was treated by intravenous antibiotics and submitted to surgery. During surgery, an inflamed and perforated post-ileal appendix (Fig. 2), with associated pericecal abscess, was found and an open appendenctomy was performed. The postoperative course was uneventful and the patient was discharged on the 5^th^ post-operative day. Pathological examination showed the presence of a non-mucinous tubulo-villous adenoma with moderate epithelial dysplasia (Fig. 3, Fig. 4) with negative resection margin. One month after surgery, the patient accepted to undergo colonoscopy without evidence of synchronous colon and rectal tumors.

## 3 Discussion

This case describes an acute appendicitis caused by a rare benign epithelial lesion. Appendiceal epithelial neoplasms are observed in 0.2–0.3% of appendectomy specimens and occur most commonly in the 5^th^–7^th^ decades of life [5], as in our case. Moreover tubulo-villous adenoma is a rare histological type of non-mucinous epithelial neoplasm which represents only 30% of all appendiceal epithelial neoplasms [6]. The great rarity of these neoplasms has been attributed to the small surface area of the appendix in relation to the remainder of the colon. The diagnosis is notoriously difficult, in fact these lesions are often asymptomatic and occasionally discovered at surgery or at pathology examination of the surgical specimen: a review of appendiceal adenomas showed that more than 93% of these benign lesions were diagnosed after an appendectomy or at autopsy, often associated with other diseases such as acute or chronic recurrent appendicitis, peritonitis from perforated appendicitis and intussusceptions [7]. A retrospective study about occult appendiceal neoplasms of 1793 appendectomies showed that patients who undergo interval appendectomies for chronic appendicitis or incidental appendectomies as part of a largen specimen (i.e. colectomy) or as a part of ovarian cancer staging, may be at higher risk of appendiceal neoplasm compared with those performed for acute appendicitis [8]. When these neoplasms have a symptomatic presentation, as in our case, the most common symptom is acute appendicitis, although in elderly patients occur only in 5–10% of cases [8]. Less common symptoms are abdominal pain, a palpable mass, gastrointestinal or genitourinary obstruction, gastrointestinal bleeding and hematuria. Sometimes the diagnosis is accidentally obtained during radiologic or endoscopic examinations performed to evaluate other conditions of the abdominal cavity [9]. Among radiological imaging, ultrasonography (US) is a valuable exam for detection of appendiceal neoplasms, but it is highly operator dependent and limited in staging of tumors, instead abdominal CT with or without intravenous and oral contrast material is the most accurate method on detection of appendiceal neoplasms, with a high sensivity of 95% [10]. In our case, unfortunately, imaging didn’t show the presence of appendiceal adenoma. Colonoscopy isn’t a useful screening tool for appendiceal neoplasm, with a sensitivity of only 11%: the difficulty of diagnosis lies in the fact that these neoplasms are small and located within the lumen of the appendix which prevents the access of the colonoscope [11]. Endoscopic diagnosis is easier to establish if the neoplasm affects the ostium of appendix and reaches the mucosa of the cecum. Given the lack of pathognomonic symptoms and specific radiological findings, several risk factors have been identified to favor an early diagnosis of appendiceal tumors among patients presenting with acute appendicitis: older age (55–65 years), abnormally duration of symptoms (>2 days) [12], absence of migratory right lower quadrant pain [13], low hematocrit (<38%) [12], outpatient steroid/immunosuppressant use and the appearance of a phlegmon on CT scan [13]. In patients presenting with appendiceal inflammatory mass the rate of incidental neoplasms is much higher, from 10 to 29% [14]. In our case the patient was elderly, had abdominal pain for more than two days without migration in the right lower quadrant and there was the presence of appendiceal phlegmon on CT scan. Since acute appendicitis could be a clinical manifestation of an appendiceal neoplasm, its treatment is debated: some surgeons suggest operative treatment, but others advocate for non-operative management of selected cases recommending follow-up imaging in attempt to capture undiagnosed neoplasms. However this option doesn’t appear adequate since benign appendiceal neoplasms, including the tubulo-villous adenoma of our case, are frequently not evidenced on imaging studies [15] and presenting a risk of malignant degeneration. To date, limited literature doesn’t allow the establishment of guidelines but only recommendations on the correct diagnostic and therapeutic treatment of this pathology. Appendectomy alone is generally considered adequate for most benign appendiceal lesions like adenoma. Right hemicolectomy with nodal dissection is recommended in case of tumor size greater than 2 cm or malignant degeneration of adenoma with mesoappendicular invasion, nodal extension, contaminated margin, lymphatic or vascular emboli [16]. Endoscopic mucosal resection of appendiceal adenoma isn’t a prudent option of treatment because it carries the risk of leaving residual tissue in the appendiceal lumen with potential malignant transformation [17]. Colonoscopy should be scheduled at 1 month after surgery in patients with already diagnosed appendiceal neoplasm, as in our case, to exclude synchronous neoplasms of colon and rectum and is recommended in the follow-up every 6 months for 2 years, followed by every 3 years, if results remain normal, to detect any recurrences or metachronous neoplasms. Yearly monitoring of carcinoembryonic antigen (CEA) levels for the first 5 years is also recommended [18].

## 4 Conclusion

Tubulo-villous adenoma of the appendix is very uncommon and mostly discovered after surgery. Preoperative diagnosis is limited because of a frequent lack of symptoms and poor diagnostic signs. Acute appendicitis is the most common presentation and treatment is in any case surgical resection.

## Sources of funding

This research did not receive any specific grant from funding agencies in the public, commercial, or not-for-profit sectors.

## Ethical approval

Ethical approval has been exempted by our institution because this is a case report and no new studies or new techniques were carried out.

## Consent

Written informed consent was obtained from the patient for publication of this case report and accompanying images.

## Author’s contribution

Giuseppe Evola: Operated on the patient, drafting the manuscript.

Giovambattista Caruso: Drafting the manuscript and literature research.

Sebastiano Caramma: Operated on the patient, literature research.

Giovanni Dapri: Revising the manuscript.

Carmela Spampinato: Literature research.

Carlo Reina: Drafting the manuscript and literature research.

Giuseppe Angelo Reina: Clinical supervision and consultation.

## Registration of research studies

This case report does not require registration as a research study.

## Guarantor

The guarantor for this case report is Giuseppe Evola.

## Provenance and peer review

Not commissioned, externally peer-reviewed.

## Declaration of Competing Interest

All the authors certify that there is no conflict of interest regarding the material discussed in the manuscript.
